# Whole-Genome Sequencing of Legionella jordanis Strains NML 060502 and NML 130005, Recovered from a Lower Respiratory Tract Infection and Water, Respectively

**DOI:** 10.1128/MRA.01537-18

**Published:** 2019-01-24

**Authors:** Anne-Marie Bernier, Kathryn Bernard

**Affiliations:** a Department of Biology, Université de Saint-Boniface, Winnipeg, Canada; b National Microbiology Laboratory (NML)—CSCHAH Site, Public Health Agency of Canada, Winnipeg, Canada; c Department of Medical Microbiology, University of Manitoba, Winnipeg, Canada; University of Maryland School of Medicine

## Abstract

Draft genome sequences of two strains of the rarely isolated organism Legionella jordanis, NML 060502 (from a patient with a lower respiratory infection) and NML 130005 (from water), were assembled and studied. Respectively, the genome sizes obtained were 2,927,328 bp and 3,101,130 bp, with G+C contents of 41.9% and 41.7%.

## ANNOUNCEMENT

Legionella jordanis, first described in 1982 after recovery from the environment (1), has also been associated with fatal pneumonia (2) but remains a rare cause of infection. The Canadian National Microbiology Laboratory (NML) has been referred only two L. jordanis isolates in the past 35 years. Strain NML 060502 was recovered from a New Brunswick patient with a lower respiratory infection (3), and strain NML 130005 was recovered from water taken from a house in Alberta during an outbreak investigation in the winter of 2013; these strains were therefore considered temporally and geographically unrelated. As the genome of L. jordanis is currently represented in the NCBI database only by ATCC 33623^T^ (GenBank accession number LNYJ00000000) (4), we provide here draft genomes for these isolates.

Bacteria were subcultured after storage at −80°C in Microbank vials (Pro Lab) from NML stocks and passed twice at 35°C on buffered charcoal yeast extract plates (BCYE; Oxoid) for 48 h in a candle jar. DNA was extracted from a loopful of plate culture using a DNA minikit (Qiagen), and paired-end whole-genome shotgun libraries were constructed using a Nextera XT library preparation kit. Samples were run separately for sequencing using a MiSeq 600-cycle kit (v3) on a MiSeq sequencer (Illumina). Read quality was assessed with FastQC v0.11.8 (http://www.bioinformatics.babraham.ac.uk/projects/fastqc/), and reads were assembled using SPAdes (v3.9.0; default settings) (5) after merging short paired-end reads with Fast Length Adjustment of SHort reads (FLASH v1.2.9; default settings) (6). Genomes were analyzed using JSpeciesWS to calculate the average nucleotide identity (ANI) values using BLAST+ (ANIb) (7). NML 060502 and NML 130005 were found to have ANIb scores greater than 99.5% similar to each other and to that of ATCC 33623^T^. The Genome-to-Genome Distance Calculator v2.1 (8) was used to estimate *in silico* DNA-DNA hybridization (*is*DDH) values between strains. Using recommended formula 2 for draft genomes, we found the two NML strains to have *is*DDH values greater than 97.3% similar to each other and to that of the type strain.

The sequencing run for NML 060502 produced 348,204 sequences of 35 to 301 bases in length, with a total of 99,624,247 bases. The draft genome of NML 060502 was comprised of 2,927,328 bp, which assembled into 30 contigs with 59× coverage, a G+C content of 41.91%, and an *N*_50_ contig length of 205,575. The genome, annotated by Prokka (version 1.13) (9), coded for 2,635 proteins, of which 74.5% were assigned to Clusters of Orthologous Groups (COGs) categories using eggNOG-Mapper (10). This genome encoded 5 rRNA genes and 41 tRNAs, and no CRISPR elements were detected.

The sequencing run for NML 130005 produced 539,938 sequencing reads (length, 35 to 301 bases), with a total of 150,406,415 bases. The draft genome of NML 130005 consisted of 3,101,130 bp, which assembled into 22 contigs with 96× coverage, a G+C content of 41.7%, and an *N*_50_ contig length of 374,282. eggNOG-Mapper assigned 74.5% of the 2,823 coding regions to COGs, and the draft genome encoded 5 rRNA genes and 41 tRNAs, with no CRISPR elements being found. Neither genome harbored intact phages, as evaluated using PHASTER (11). Genes homologous to the type IVB Icm/Dot secretion system required for intracellular growth were identified in the three genomes, consistent with their presence in Legionella species (4).

### Data availability.

Draft genome sequences of Legionella jordanis strains NML 060502 and NML 130005 were deposited at DDBJ/ENA/GenBank under the accession numbers RDQQ00000000 and RDQP00000000, respectively. The versions described in this paper are RDQQ01000000 and RDQP01000000, and raw reads were assigned the accession numbers SRX4988200 and SRX4988201 in the NCBI Sequence Read Archive.
